# 4-[(2-Bromo­benzyl­idene)amino]-3-(pyridin-4-yl)-1*H*-1,2,4-triazole-5(4*H*)-thione

**DOI:** 10.1107/S1600536812024439

**Published:** 2012-06-02

**Authors:** Wei Gao, Xian Li, Xin-Ling Wang, Jing Yang, Xue-Fen Wu

**Affiliations:** a School of Pharmacy, Henan University of Traditional Chinese Medicine, Zhengzhou 450008, People’s Republic of China

## Abstract

In the title compound, C_14_H_10_BrN_5_S, the dihedral angle between the triazole ring and the pyridine and bromo­benzene rings are 26.42 (13) and 6.28 (13)°, respectively. The molecule exists as a thione in the solid state. In the crystal, mol­ecules are linked by N—H⋯N hydrogen bonds, generating [010] *C*(8) chains.

## Related literature

For related structures, see: Zou *et al.* (2008 ▶); Kashaev *et al.* (2010 ▶); Liu & Liu (2011 ▶); Liu, Pan, Weng, Tan *et al.* (2012 ▶); Liu, Tan, Weng & Liu (2012 ▶); Tan *et al.* (2012 ▶).
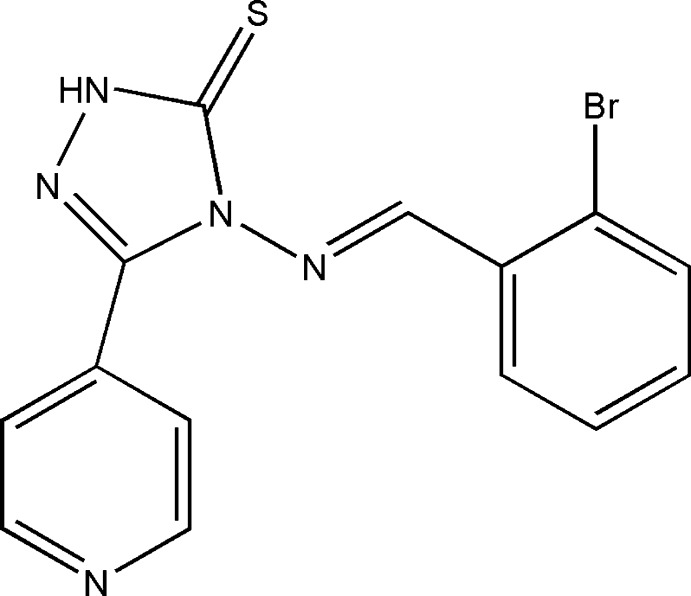


## Experimental

### 

#### Crystal data

C_14_H_10_BrN_5_S
*M* *_r_* = 360.24
Monoclinic, 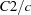

*a* = 10.406 (3) Å
*b* = 17.299 (5) Å
*c* = 15.858 (4) Å
β = 103.719 (5)°
*V* = 2773.3 (13) Å^3^
*Z* = 8
Mo *K*α radiation
μ = 3.12 mm^−1^
*T* = 113 K
0.20 × 0.18 × 0.12 mm

#### Data collection

Rigaku Saturn CCD diffractometer
Absorption correction: multi-scan (*CrystalClear*; Rigaku/MSC, 2005 ▶) *T*_min_ = 0.575, *T*_max_ = 0.706
14119 measured reflections
3293 independent reflections
2565 reflections with *I* > 2σ(*I*)
*R*_int_ = 0.036

#### Refinement

*R*[*F* ^2^ > 2σ(*F* ^2^)] = 0.030
*wR*(*F* ^2^) = 0.087
*S* = 1.08
3293 reflections
194 parameters
1 restraint
H atoms treated by a mixture of independent and constrained refinement
Δρ_max_ = 0.54 e Å^−3^
Δρ_min_ = −0.42 e Å^−3^

### 

Data collection: *CrystalClear* (Rigaku/MSC, 2005 ▶); cell refinement: *CrystalClear*; data reduction: *CrystalClear*; program(s) used to solve structure: *SHELXS97* (Sheldrick, 2008 ▶); program(s) used to refine structure: *SHELXL97* (Sheldrick, 2008 ▶); molecular graphics: *SHELXTL* (Sheldrick, 2008 ▶); software used to prepare material for publication: *SHELXL97*.

## Supplementary Material

Crystal structure: contains datablock(s) global, I. DOI: 10.1107/S1600536812024439/hb6820sup1.cif

Structure factors: contains datablock(s) I. DOI: 10.1107/S1600536812024439/hb6820Isup2.hkl

Supplementary material file. DOI: 10.1107/S1600536812024439/hb6820Isup3.cml

Additional supplementary materials:  crystallographic information; 3D view; checkCIF report

## Figures and Tables

**Table 1:** Hydrogen-bond geometry (Å, °)

*D*—H⋯*A*	*D*—H	H⋯*A*	*D*⋯*A*	*D*—H⋯*A*
N3—H3⋯N1^i^	0.90 (1)	1.95 (1)	2.821 (3)	163 (3)

Symmetry code: (i) 
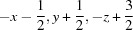
.

**Fig. 1.**
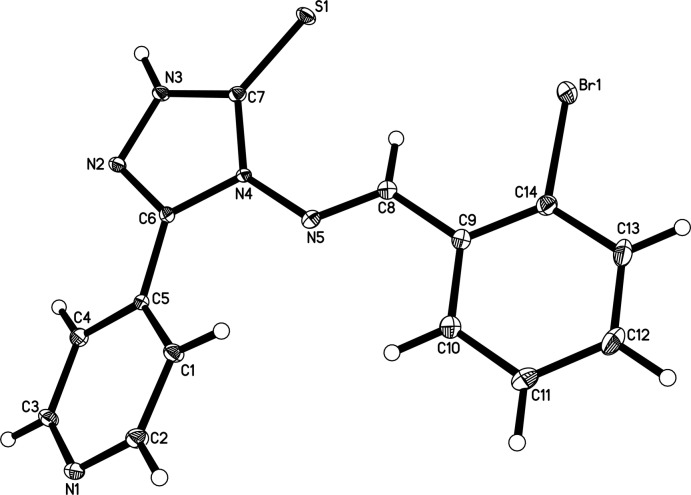
The molecular structure of (I). Displacement ellipsoids are drawn at the 30% probability level and H atoms are shown as small spheres of arbitrary radii.

**Fig. 2.**
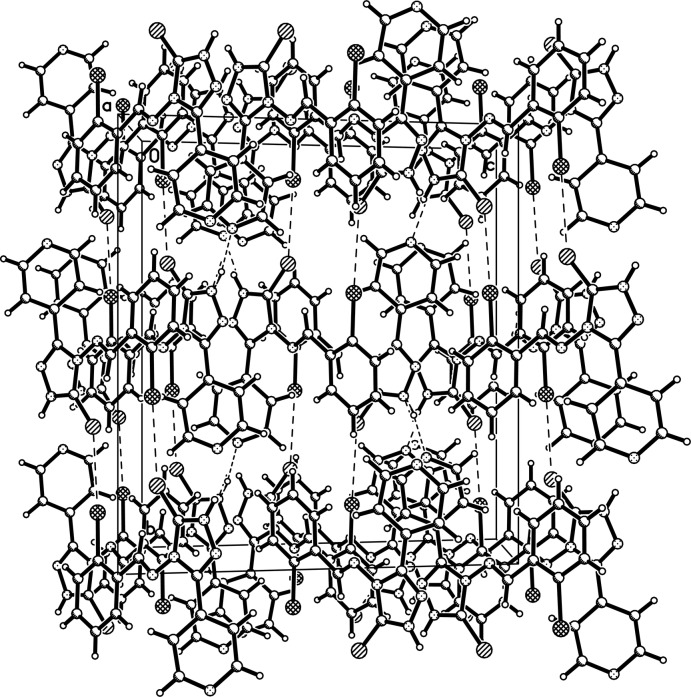
The crystal packing for (I).

## supplementary crystallographic information

### Comment

Single-crystal X-ray diffraction analysis reveals that the title compound crystallizes in the monoclinic space group *C*2/*c*. As shown in Fig. 1, the dihedral angle between the pyridyl and triazole rings is 153.6 °. As shown in Fig. 2, the crystal structure is stabilized by intermolecular hydrogen bonds N—H···N.

### Experimental

In round bottom flask, 4-pyridine carboxylic acid (0.01 mol) and hydrazine hydrate 99% (0.01 mol) were taken along with alcohol and the mixture was refluxed for 4 h. Then from the reaction mixture alcohol was removed under reduced pressure. Solid residue was obtained, recrystallized from ethanol. In a 250 ml round bottom flask, 4-pyridine hydrazide (0.075 mol) was taken. To this a solution of potassium hydroxide (0.075 mol) in 100 ml of absolute alcohol and carbon disulfide were added agitated for overnight. The reaction mixture was diluted with 200 ml of dry ether. The solid obtained was 15.05 g (80%). It was filtered and washed with dry ether. A mixture of potassium-pyridine-dithiocarbazate (0.1 mol) and hydrazine hydrate 5 ml (0.1 mol) was refluxed for 2 h with occasional shaking and the solution was poured into the cold water. The mixture was acidified with hydrochloric acid. The precipitate obtained was filtered, dried and recrystallized by using alcohol. A mixture of 5-pyridine-4-amino-3-mercapto-4(H)-1,2,4-triazole (0.01 mol) taken with 2-bromobenzaldehyde (0.01 mol) and concentrated sulfuric acid (0.5 ml) in ethanol 100 ml. The mixture was refluxed on water bath for several hours with TLC monitoring. The solid was obtained on cooling the mixture and poured in cold water was afforded the the title compound. Colourless prisms were grown from ethanol solution.

### Refinement

All the H atoms were positioned geometrically (C—H = 0.93–0.97 Å) and refined as riding with *U*_iso_(H) = 1.2*U*_eq_(C) or 1.5*U*_eq_(methyl C).

### Crystal data

**Table:**

C_14_H_10_BrN_5_S	*F*(000) = 1440
*M**_r_* = 360.24	*D*_x_ = 1.726 Mg m^−^^3^
Monoclinic, *C*2/*c*	Mo *K*α radiation, λ = 0.71073 Å
*a* = 10.406 (3) Å	Cell parameters from 5149 reflections
*b* = 17.299 (5) Å	θ = 2.3–27.9°
*c* = 15.858 (4) Å	µ = 3.12 mm^−^^1^
β = 103.719 (5)°	*T* = 113 K
*V* = 2773.3 (13) Å^3^	Prism, colorless
*Z* = 8	0.20 × 0.18 × 0.12 mm

### Data collection

**Table:**

Rigaku Saturn CCD diffractometer	3293 independent reflections
Radiation source: rotating anode	2565 reflections with *I* > 2σ(*I*)
Multilayer monochromator	*R*_int_ = 0.036
Detector resolution: 14.63 pixels mm^-1^	θ_max_ = 27.9°, θ_min_ = 2.3°
ω and φ scans	*h* = −13→13
Absorption correction: multi-scan (*CrystalClear*; Rigaku/MSC, 2005)	*k* = −22→22
*T*_min_ = 0.575, *T*_max_ = 0.706	*l* = −20→20
14119 measured reflections	

### Refinement

**Table:**

Refinement on *F*^2^	Primary atom site location: structure-invariant direct methods
Least-squares matrix: full	Secondary atom site location: difference Fourier map
*R*[*F*^2^ > 2σ(*F*^2^)] = 0.030	Hydrogen site location: inferred from neighbouring sites
*wR*(*F*^2^) = 0.087	H atoms treated by a mixture of independent and constrained refinement
*S* = 1.08	*w* = 1/[σ^2^(*F*_o_^2^) + (0.0476*P*)^2^ + 0.9724*P*] where *P* = (*F*_o_^2^ + 2*F*_c_^2^)/3
3293 reflections	(Δ/σ)_max_ = 0.003
194 parameters	Δρ_max_ = 0.54 e Å^−^^3^
1 restraint	Δρ_min_ = −0.42 e Å^−^^3^

### Special details

**Table:**

Geometry. All e.s.d.'s (except the e.s.d. in the dihedral angle between two l.s. planes) are estimated using the full covariance matrix. The cell e.s.d.'s are taken into account individually in the estimation of e.s.d.'s in distances, angles and torsion angles; correlations between e.s.d.'s in cell parameters are only used when they are defined by crystal symmetry. An approximate (isotropic) treatment of cell e.s.d.'s is used for estimating e.s.d.'s involving l.s. planes.
Refinement. Refinement of *F*^2^ against ALL reflections. The weighted *R*-factor *wR* and goodness of fit *S* are based on *F*^2^, conventional *R*-factors *R* are based on *F*, with *F* set to zero for negative *F*^2^. The threshold expression of *F*^2^ > σ(*F*^2^) is used only for calculating *R*-factors(gt) *etc*. and is not relevant to the choice of reflections for refinement. *R*-factors based on *F*^2^ are statistically about twice as large as those based on *F*, and *R*- factors based on ALL data will be even larger.

### Fractional atomic coordinates and isotropic or equivalent isotropic displacement parameters (Å^2^)

**Table:**

	*x*	*y*	*z*	*U*_iso_*/*U*_eq_	
Br1	0.42237 (3)	1.115195 (15)	1.078191 (18)	0.02495 (10)	
S1	0.04950 (6)	1.18904 (3)	0.90838 (4)	0.01831 (15)	
N1	−0.2275 (2)	0.76260 (12)	0.76957 (13)	0.0170 (4)	
N2	−0.1987 (2)	1.05219 (11)	0.75708 (13)	0.0135 (4)	
N3	−0.1441 (2)	1.12183 (11)	0.78721 (13)	0.0135 (4)	
N4	−0.02113 (18)	1.03665 (11)	0.86394 (12)	0.0110 (4)	
N5	0.0722 (2)	0.99264 (12)	0.92068 (13)	0.0165 (4)	
C1	−0.1154 (3)	0.86370 (13)	0.86163 (16)	0.0157 (5)	
H1	−0.0638	0.8787	0.9170	0.019*	
C2	−0.1571 (3)	0.78812 (14)	0.84586 (17)	0.0185 (5)	
H2	−0.1341	0.7521	0.8923	0.022*	
C3	−0.2600 (2)	0.81462 (13)	0.70517 (17)	0.0178 (5)	
H3A	−0.3098	0.7975	0.6502	0.021*	
C4	−0.2249 (2)	0.89158 (13)	0.71470 (16)	0.0149 (5)	
H4	−0.2511	0.9263	0.6673	0.018*	
C5	−0.1507 (2)	0.91779 (13)	0.79426 (16)	0.0123 (5)	
C6	−0.1234 (2)	1.00094 (13)	0.80478 (15)	0.0111 (4)	
C7	−0.0371 (2)	1.11627 (13)	0.85297 (15)	0.0125 (5)	
C8	0.1806 (2)	1.02341 (14)	0.96104 (16)	0.0177 (5)	
H8	0.1978	1.0765	0.9533	0.021*	
C9	0.2775 (2)	0.97532 (14)	1.01938 (15)	0.0158 (5)	
C10	0.2607 (3)	0.89478 (14)	1.02189 (18)	0.0203 (5)	
H10	0.1857	0.8714	0.9846	0.024*	
C11	0.3519 (3)	0.84900 (15)	1.07796 (18)	0.0234 (6)	
H11	0.3396	0.7946	1.0786	0.028*	
C12	0.4613 (3)	0.88260 (16)	1.13319 (19)	0.0257 (6)	
H12	0.5230	0.8511	1.1721	0.031*	
C13	0.4808 (3)	0.96139 (16)	1.13185 (17)	0.0234 (6)	
H13	0.5559	0.9843	1.1694	0.028*	
C14	0.3892 (2)	1.00692 (14)	1.07490 (16)	0.0175 (5)	
H3	−0.175 (3)	1.1661 (12)	0.761 (2)	0.049 (10)*	

### Atomic displacement parameters (Å^2^)

**Table:**

	*U*^11^	*U*^22^	*U*^33^	*U*^12^	*U*^13^	*U*^23^
Br1	0.01786 (15)	0.02020 (14)	0.03306 (18)	−0.00296 (10)	−0.00141 (11)	−0.00368 (11)
S1	0.0216 (3)	0.0097 (3)	0.0195 (3)	−0.0023 (2)	−0.0033 (3)	−0.0012 (2)
N1	0.0181 (11)	0.0111 (9)	0.0198 (12)	−0.0002 (8)	0.0006 (9)	−0.0007 (8)
N2	0.0148 (10)	0.0094 (9)	0.0149 (10)	−0.0007 (7)	0.0005 (8)	−0.0008 (8)
N3	0.0163 (10)	0.0082 (9)	0.0140 (11)	0.0002 (8)	−0.0005 (9)	0.0015 (8)
N4	0.0113 (9)	0.0078 (9)	0.0124 (10)	0.0010 (7)	−0.0001 (8)	0.0001 (8)
N5	0.0159 (10)	0.0134 (10)	0.0185 (11)	0.0026 (8)	0.0007 (9)	0.0012 (8)
C1	0.0190 (12)	0.0125 (10)	0.0135 (12)	−0.0014 (9)	−0.0003 (10)	0.0000 (9)
C2	0.0223 (13)	0.0137 (11)	0.0173 (13)	0.0006 (10)	0.0001 (11)	0.0031 (10)
C3	0.0193 (12)	0.0131 (11)	0.0187 (13)	−0.0028 (9)	0.0001 (10)	−0.0030 (10)
C4	0.0161 (12)	0.0144 (11)	0.0129 (12)	0.0004 (9)	0.0007 (10)	0.0025 (9)
C5	0.0095 (11)	0.0098 (10)	0.0168 (13)	0.0002 (8)	0.0017 (10)	−0.0006 (9)
C6	0.0108 (11)	0.0115 (10)	0.0106 (11)	−0.0002 (8)	0.0016 (9)	−0.0008 (9)
C7	0.0140 (11)	0.0098 (10)	0.0139 (12)	0.0003 (9)	0.0038 (10)	0.0015 (9)
C8	0.0162 (12)	0.0149 (11)	0.0206 (13)	0.0005 (9)	0.0017 (11)	−0.0011 (10)
C9	0.0143 (11)	0.0170 (11)	0.0159 (13)	0.0004 (9)	0.0032 (10)	0.0005 (10)
C10	0.0167 (12)	0.0197 (13)	0.0237 (15)	−0.0002 (10)	0.0032 (11)	−0.0007 (10)
C11	0.0266 (14)	0.0180 (13)	0.0257 (15)	0.0048 (11)	0.0064 (12)	0.0062 (11)
C12	0.0244 (14)	0.0285 (14)	0.0222 (15)	0.0100 (12)	0.0013 (12)	0.0065 (12)
C13	0.0157 (13)	0.0313 (15)	0.0207 (14)	0.0040 (11)	−0.0005 (11)	0.0001 (12)
C14	0.0169 (12)	0.0175 (12)	0.0183 (13)	0.0019 (10)	0.0043 (11)	−0.0003 (10)

### Geometric parameters (Å, º)

**Table:**

Br1—C14	1.903 (3)	C3—H3A	0.9500
S1—C7	1.671 (2)	C4—C5	1.390 (3)
N1—C2	1.332 (3)	C4—H4	0.9500
N1—C3	1.343 (3)	C5—C6	1.468 (3)
N2—C6	1.300 (3)	C8—C9	1.457 (3)
N2—N3	1.369 (3)	C8—H8	0.9500
N3—C7	1.336 (3)	C9—C14	1.393 (3)
N3—H3	0.896 (10)	C9—C10	1.406 (3)
N4—N5	1.385 (3)	C10—C11	1.385 (4)
N4—C6	1.386 (3)	C10—H10	0.9500
N4—C7	1.393 (3)	C11—C12	1.389 (4)
N5—C8	1.274 (3)	C11—H11	0.9500
C1—C2	1.382 (3)	C12—C13	1.379 (4)
C1—C5	1.402 (3)	C12—H12	0.9500
C1—H1	0.9500	C13—C14	1.391 (3)
C2—H2	0.9500	C13—H13	0.9500
C3—C4	1.380 (3)		
			
C2—N1—C3	117.0 (2)	N4—C6—C5	127.6 (2)
C6—N2—N3	104.72 (19)	N3—C7—N4	102.8 (2)
C7—N3—N2	114.15 (19)	N3—C7—S1	127.00 (17)
C7—N3—H3	125 (2)	N4—C7—S1	130.19 (19)
N2—N3—H3	121 (2)	N5—C8—C9	118.5 (2)
N5—N4—C6	120.17 (18)	N5—C8—H8	120.7
N5—N4—C7	132.0 (2)	C9—C8—H8	120.7
C6—N4—C7	107.83 (19)	C14—C9—C10	117.5 (2)
C8—N5—N4	119.8 (2)	C14—C9—C8	121.7 (2)
C2—C1—C5	118.6 (2)	C10—C9—C8	120.8 (2)
C2—C1—H1	120.7	C11—C10—C9	120.9 (3)
C5—C1—H1	120.7	C11—C10—H10	119.5
N1—C2—C1	124.0 (2)	C9—C10—H10	119.5
N1—C2—H2	118.0	C10—C11—C12	120.0 (3)
C1—C2—H2	118.0	C10—C11—H11	120.0
N1—C3—C4	123.4 (2)	C12—C11—H11	120.0
N1—C3—H3A	118.3	C13—C12—C11	120.4 (3)
C4—C3—H3A	118.3	C13—C12—H12	119.8
C3—C4—C5	119.4 (2)	C11—C12—H12	119.8
C3—C4—H4	120.3	C12—C13—C14	119.3 (3)
C5—C4—H4	120.3	C12—C13—H13	120.4
C4—C5—C1	117.6 (2)	C14—C13—H13	120.4
C4—C5—C6	118.3 (2)	C13—C14—C9	121.9 (2)
C1—C5—C6	124.0 (2)	C13—C14—Br1	116.6 (2)
N2—C6—N4	110.48 (19)	C9—C14—Br1	121.48 (19)
N2—C6—C5	121.9 (2)		
			
C6—N2—N3—C7	−0.5 (3)	N2—N3—C7—N4	1.4 (3)
C6—N4—N5—C8	164.5 (2)	N2—N3—C7—S1	−176.78 (17)
C7—N4—N5—C8	−16.5 (3)	N5—N4—C7—N3	179.1 (2)
C3—N1—C2—C1	−0.7 (4)	C6—N4—C7—N3	−1.7 (2)
C5—C1—C2—N1	1.2 (4)	N5—N4—C7—S1	−2.8 (4)
C2—N1—C3—C4	−0.3 (4)	C6—N4—C7—S1	176.36 (18)
N1—C3—C4—C5	0.7 (4)	N4—N5—C8—C9	−179.17 (19)
C3—C4—C5—C1	−0.1 (3)	N5—C8—C9—C14	−170.6 (2)
C3—C4—C5—C6	−175.3 (2)	N5—C8—C9—C10	9.0 (4)
C2—C1—C5—C4	−0.8 (3)	C14—C9—C10—C11	0.3 (4)
C2—C1—C5—C6	174.1 (2)	C8—C9—C10—C11	−179.3 (2)
N3—N2—C6—N4	−0.7 (2)	C9—C10—C11—C12	0.5 (4)
N3—N2—C6—C5	178.5 (2)	C10—C11—C12—C13	−0.8 (4)
N5—N4—C6—N2	−179.16 (18)	C11—C12—C13—C14	0.4 (4)
C7—N4—C6—N2	1.6 (3)	C12—C13—C14—C9	0.4 (4)
N5—N4—C6—C5	1.7 (3)	C12—C13—C14—Br1	179.2 (2)
C7—N4—C6—C5	−177.5 (2)	C10—C9—C14—C13	−0.8 (4)
C4—C5—C6—N2	23.8 (3)	C8—C9—C14—C13	178.8 (2)
C1—C5—C6—N2	−151.0 (2)	C10—C9—C14—Br1	−179.52 (18)
C4—C5—C6—N4	−157.1 (2)	C8—C9—C14—Br1	0.1 (3)
C1—C5—C6—N4	28.0 (4)		

### Hydrogen-bond geometry (Å, º)

**Table:**

*D*—H···*A*	*D*—H	H···*A*	*D*···*A*	*D*—H···*A*
N3—H3···N1^i^	0.90 (1)	1.95 (1)	2.821 (3)	163 (3)

Symmetry code: (i) −*x*−1/2, *y*+1/2, −*z*+3/2.
